# A signal recognition particle receptor gene from the sea cucumber, *Apostichopus japonicus*

**DOI:** 10.1038/s41598-023-50320-z

**Published:** 2023-12-27

**Authors:** Jian Zhang, Zhihui Sun, Weiyi Su, Zengdong Wang, Weihan Meng, Yaqing Chang

**Affiliations:** 1 School of Life Science, Liaoning Normal University, Dalian, 116029 China; 2 Key Laboratory of Mariculture & Stock Enhancement in North China’s Sea, Ministry of Agriculture and Rural Affairs, Dalian Ocean University, Dalian, 116023 China; 3 Shandong Anyuan Aquaculture Co. Ltd, Yantai, 264000 China

**Keywords:** RNAi, Transcription

## Abstract

The signal recognition particle (SRP) system delivers approximately 30% of the proteome to the endoplasmic reticulum (ER) membrane. SRP receptor alpha (SRα) binds to SRP for targeting nascent secreted proteins to the ER membrane in eukaryotic cells. In this study, the *SRα* homologous gene was identified in the sea cucumber, *Apostichopus japonicus* (*AjSRα*). *AjSRα* codes for 641 amino acids and has 54.94% identity with its mammalian homologs. Like mammalian *SRα*, it is expected to contain the SRP-alpha N domain, SRP54_N domain, and SRP54 domain. In addition, *AjSRα* is ubiquitously expressed in adult tissues and exhibits a sexually dimorphic expression pattern, with significantly higher expression in ovaries compared to testes. As a maternal factor, *AjSRα* can be continuously detected during embryonic development. Importantly, we first attempted to investigate its function by using lentiviral vectors for delivering *SRα* gene-specific shRNA, and we revealed that lentiviral vectors do not induce an upregulation of immune-related enzymes in sea cucumbers. However, compared to the dsRNA-based RNA interference (RNAi) method, lentivirus-mediated RNAi caused dynamic changes in gene expression at a later time. This study supplied the technical support for studying the functional mechanism of *SRα* in sea cucumbers.

## Introduction

Delivering cytosolically synthesized proteins efficiently and accurately to different subcellular organelles is a crucial issue for every living cell^1^. The signal recognition particle (SRP) and its receptor (SR) are an essential and universally conserved machinery that targets nascent secretory proteins to the plasma membrane^2–4^. In eukaryotic cells, SRP is composed of 7S RNA and six different polypeptides (SRP9, SRP14, SRP19, SRP54, SRP68, and SRP72)^5^. Its receptor consists of a heterodimer α-subunit (SRα) and a eukaryotic-specific β-subunit (SRβ)^6,7^. In the SRP system, SRP binds to the nascent polypeptide on the ribosome to form a ribosome nascent chain (RNC)-SRP complex. Subsequently, SRP interacts with the SRα to facilitate deliver the RNC-SRP complex to the endoplasmic reticulum^5,8^. Interactions between SRP and SRP receptor lead to transfer of paused ribosomes to the translocon (brown cylinder) followed by release of SRP and continuation of protein resynthesis. In yeast (*Saccharomyces cerevisiae*), *SRα* is essential for cell growth, disruption of the homologous gene *SRP101* led to a six-fold decreased in cell growth rate^9^. In vertebrates, *SRα* is associated with immune processes. For example, in humans (Homo sapiens), *SRα* mutations increase susceptibility to apoptosis and led to defects in the development of neutrophil granulocytes^10^. In zebrafish (*Danio rerio*), knockout of the *SRα* cause developmental deformities and high embryonic lethality. Specific knockdown of *SRα* in neutrophils led to a significant decrease in neutrophil abundance^10^. In darkbarbel catfish (*Pelteobagrus vachelli*), *SRα* expression is significantly upregulated when the fish are infected with edwardsiella (*Edwardsiella ictaluri)*^11^. Furthermore, in mouses (*Mus musculus*), *SRα* is involved in glucose regulation in pancreatic cells, and may be a limiting factor in insulin synthesis in the glucose response^12^. However, studies on *SRα* have been rarely reported in invertebrates, especially in deuterostomes that diverged early such as echinoderms.

Sea cucumber is an ancient phylum of marine invertebrates, which belongs to Echinodermata. Sea cucumbers are widely distributed from the shore to the abyss, in some regions, they even account for nearly 80% of the total benthic invertebrate biomass^13^. Due to its high nutrient value and potent medicinal properties, the sea cucumber *Apostichopus japonicus* is an economically important mariculture species in the western North Pacific Ocean^14,15^. In addition to the extremely high economic value, sea cucumbers possess numerous unique biological characteristics, such as immunity to senescence and cancer^16,17^, and the natural ability to regenerate internal organs and body parts^18^. Hence, sea cucumbers, especially *A. japonicus*, have been widely used as primary models to study regeneration, evolution, and alleviate the symptoms of aging^13,19,20^. However, studies of sea cucumbers have been dramatically hampered due to a lack of efficient tools for gene function analyses, leading to significant challenges in understanding their genetic mechanisms and biological processes.

In previous studies, the injection of small interfering RNA (siRNA) or double-stranded RNA (dsRNA) into the coelom or muscle to create non-targeted knock-down lines had been widely used in non-model invertebrate species, including *A. japonicus*^21–23^, sea urchin (*Mesocentrotus nudus*)^24^, and Zhikong scallop (*Chlamys farreri*)^25^. In most cases, although RNA interference (RNAi) caused dynamic changes of genes expression in related biology signalling pathways, no obvious histological or phenotypic changes were found. Interestingly, a loss-of-function mutant of red eared slider turtle (*Trachemys scriptaelegans*) had been established by injecting lentivirus carrying epigenetic regulator *Kdm6b* specific short hairpin RNA (shRNA) into embryos at stage 13, and finally reveals that *Kdm6b* is essential to activate the male sex determination pathway^26,27^. Therefore, it is of great practical value to develop biotechnologies for gene function analyses by injecting lentivirus carrying target gene specific shRNA into the adult sea cucumber.

In this study, the *SRα* gene was identified, and its molecular structure and expression patterns were also characterized in *A. japonicus*. Importantly, we attempted to investigate its function by injecting lentivirus carrying *SRα* gene-specific shRNA into the adult sea cucumber coelom. Moreover, we compared the efficiency of gene knockdown using lentivirus-mediated RNAi and dsRNA-based RNAi. This study supplied the technical support for study functional mechanism of *SRα* in sea cucumber.

## Results

### Characterization of *SRα* homologous gene in *A. japonicus*

**Figure 1:**
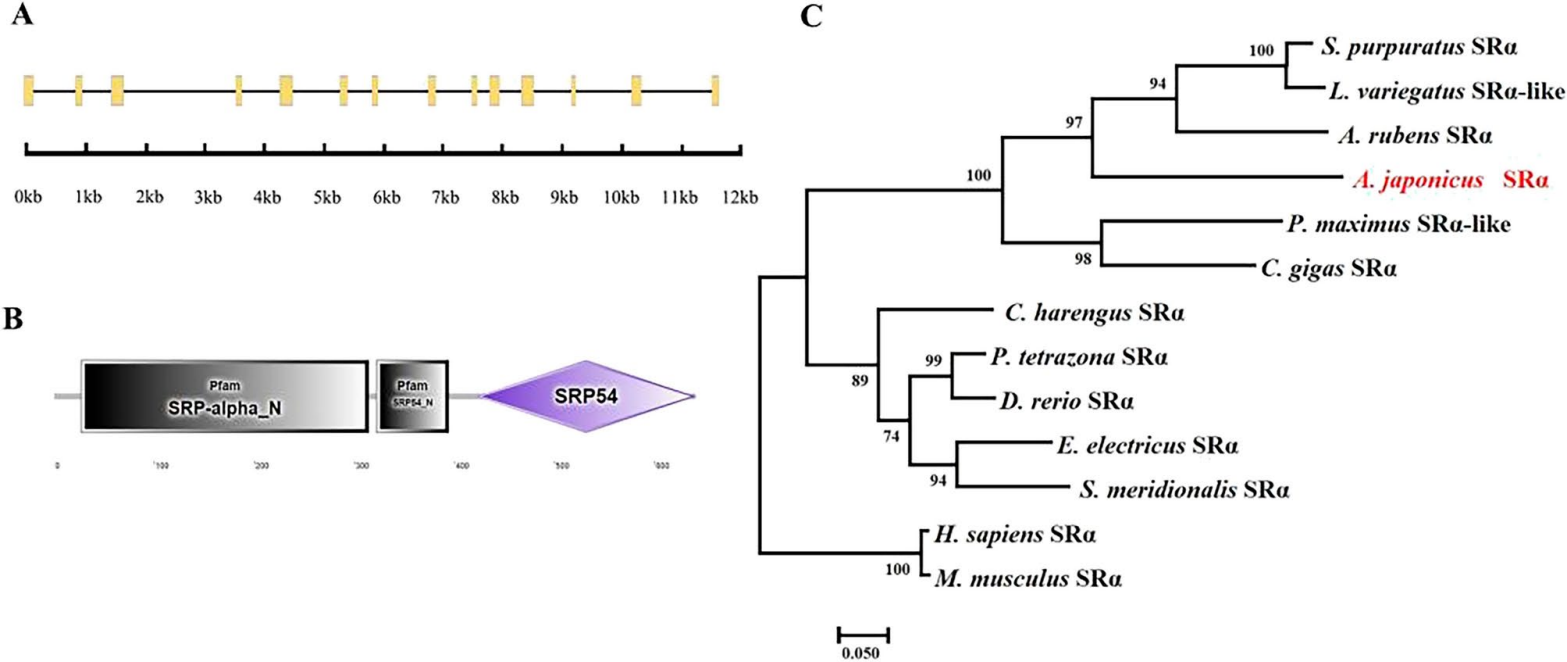
Sequence analysis of *SRα* homologous gene in *A. japonicus*. (**A**) The exon/intron structures of the *AjSRα*. The exons are indicated by yellow boxes, and black lines represent introns. (**B**) The domains structures of the *Aj*SRα. (**C**) Phylogenetic analysis of the SRα in *A. japonicus*. The phylogenetic tree was constructed with maximum likelihood analysis (1000 replicates). *A. japonicus* SRα is marked in red font.

A *SRα* transcript was cloned from the *A. japonicus* ovary cDNA library by 5’ and 3’ RACE PCR. The *SRα* cDNA is 2746 bp in length, including a 1926 bp ORF, a 777 bp 3’ untranslated region (UTR), and a short 5’ UTR that only 43 bp (GenBank: OR469739). The alignment of the cDNA sequence with the genomic sequence indicates that the *SRα* gene contains 14 exons and 13 introns (Fig. 1A). Specifically, *A. japonicus* SRα (*Aj*SRa) is a modular protein consisting of three domains, including SRP-alpha_N domain (aa 27-310), SRP54_N domain (aa 322-394) and SRP54 domain (aa 423-640) (Fig. 1B). The identity between the amino acid sequence of *Aj*SRα and other eukaryotes SRα varied from 49.19% (for the fish, *S.meridionalis*) to 58.14% (for the sea urchin, *L.variegatus*) (Figure S1). Additionally, the three domains were highly conserved, with the average identities were about 34.29% (SRP-alpha_N domain), 66.18% (SRP54_N domain), and 81.01% (SRP54 domain), respectively (Figure S2). The major differences between *Aj*SRα and other eukaryotes SRα homeologs existed in the SRP-alpha_N domain. Phylogenetic analysis showed that *Aj*SRα was clustered with SRα homologs from echinoderm species (Fig. 1C), and the topology of clades is basically consistent with the known taxonomic relationships among the analysed species.

### Expression analysis of *SRα* homologous gene in *A. japonicus*

**Figure 2:**
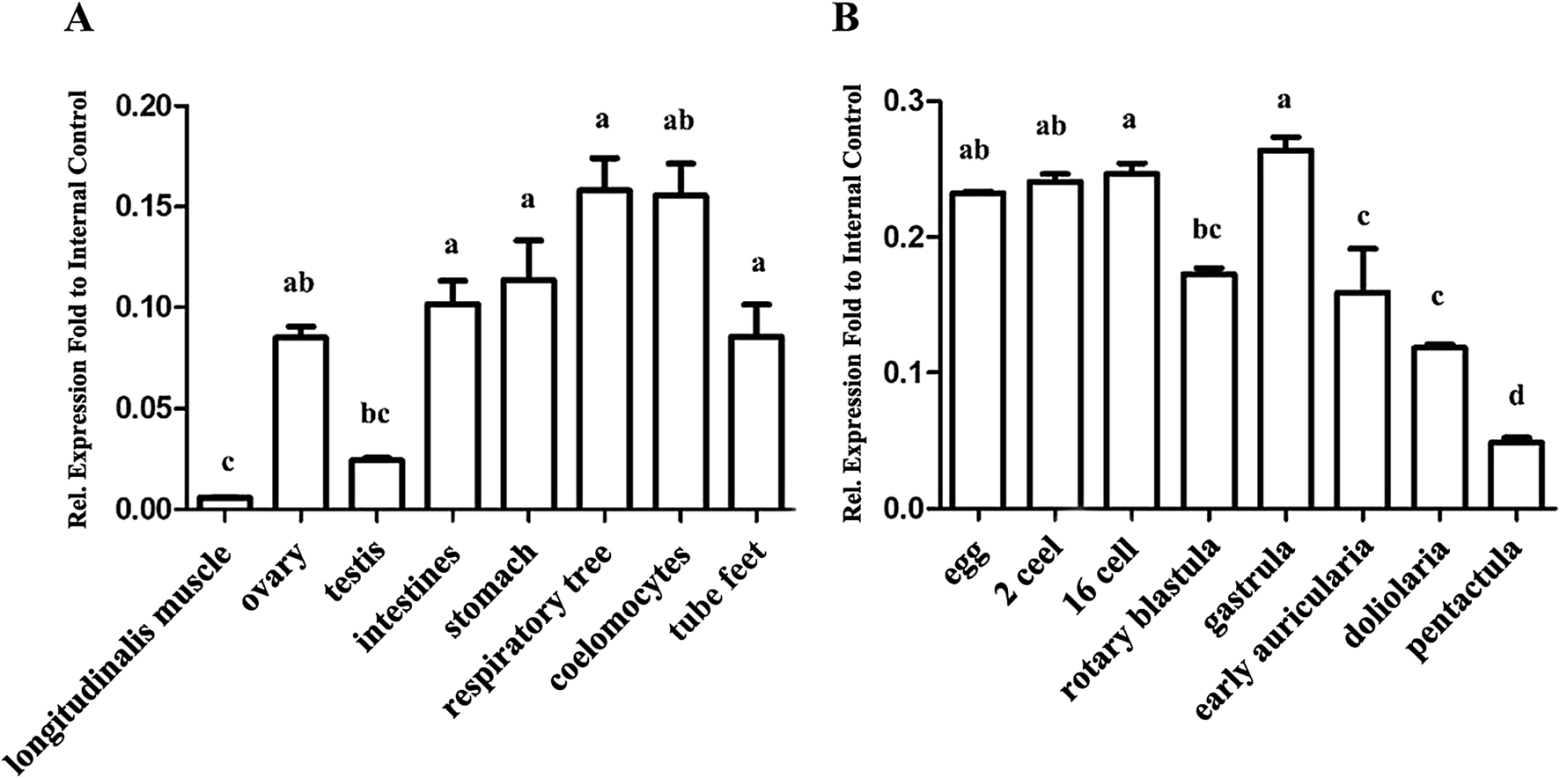
Divergent expression pattern of *AjSRα* in adult tissues and embryogenesis. (**A**) Expression pattern of *AjSRα* mRNA in different adult tissues. (**B**) Expression pattern of *AjSRα* mRNA during embryogenesis. The expression levels of *AjSRα* were normalized as a ratio of *NADH* mRNA detected in the same sample. Each bar represents mean ± standard deviation (SD) (n = 3). Different letters indicate significant differences (*p* ≤ 0.0017). Identical letters indicate no significant difference.

The distribution of *SRα* mRNAs in adult tissues and embryogenesis were detected. As shown in Fig. 2A, *AjSRα* is ubiquitously expressed in all tissues, and abundant *SRα* transcripts were amplified from analysed tissues except for longitudinal is muscle, indicating its extensive and important roles in the protein targeting pathway. Interestingly, *AjSRα* exhibited a sexually dimorphic expression pattern with remarkably higher in the ovary against the testis. Considering *AjSRα* is abundantly expressed in ovaries, we further investigated its dynamics transcripts level in embryogenesis. As expected, *AjSRα* is a maternal factor and is highly expressed from the fertilized egg to pentactula stage (Fig. 2B).

### Generation and activity testing of LV-*AjSRα*-shRNA

**Figure 3:**
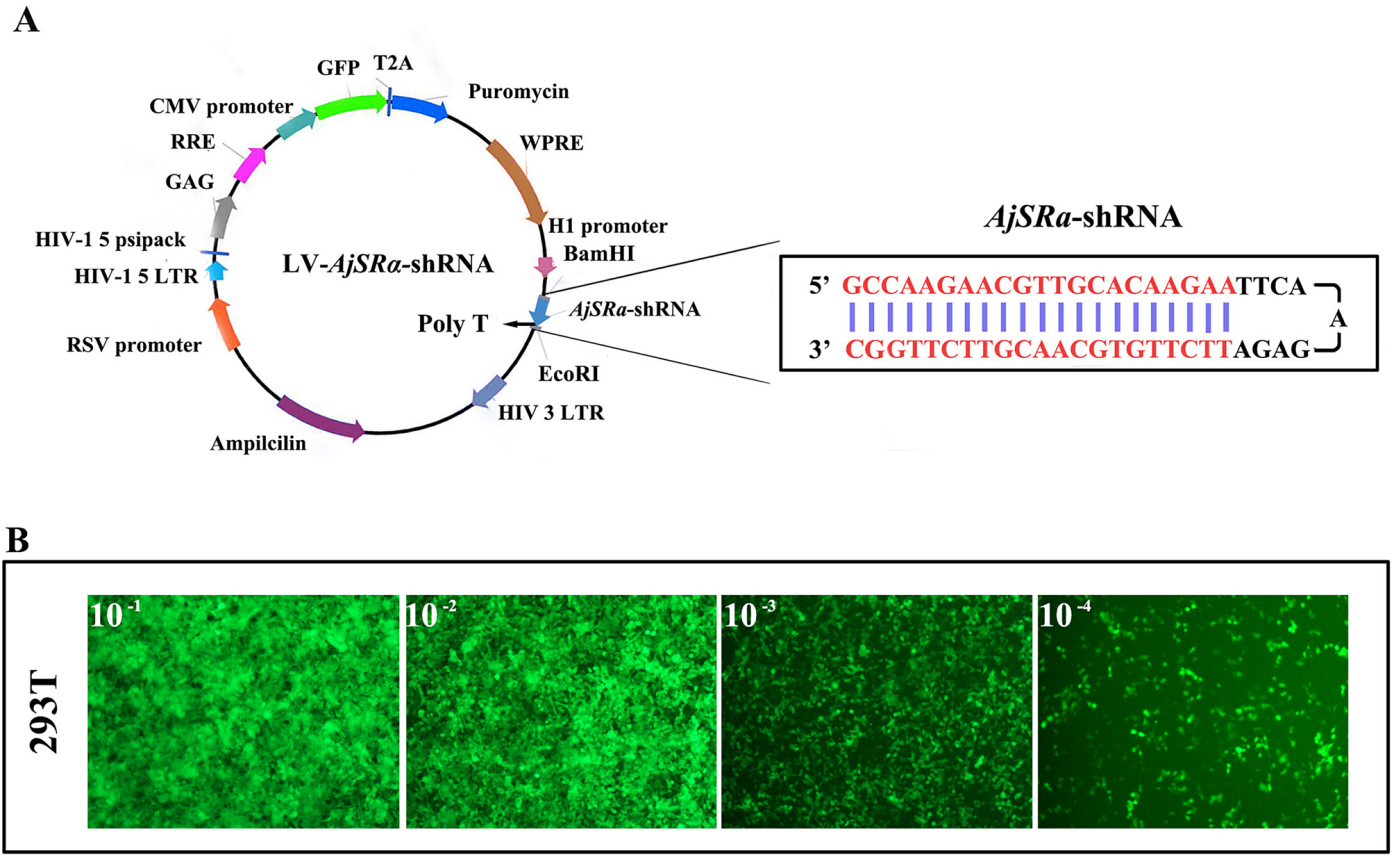
Deliver *AjSRα*-shRNA by lentivirus vector. (**A**) Plasmid diagram of LV- *AjSRα*-shRNA, *BamH I* and* EcoRI* were the cloning sites. (**B**) GFP expression after the infection of HEK293T cells with different concentrations of LV- *AjSRα*-shRNA.

To explore the functional role of *SRα* in various adult tissues of *A. japonicus*, the shRNA specific to *SRα* was cloned into the lentivirus plasmids LV3 (pGLVH1/GFP-Puro) (Fig. 3A) and co-transfected with three packaging plasmids into HEK293T cells to generate lentivirus (LV). Subsequently, the virus of LV-*AjSRα*-shRNA was used to transduce HEK293T cells, and abundant green fluorescence was detected in HEK293T cells (Fig. 3B), indicating that the LV-*AjSRα*-shRNA virus had been successfully and efficiently transduced into HEK293T cells. In addition, significantly green fluorescence can be detected in the intestines and ovary tissues after injecting the LV*-AjSRα*-shRNA virus into the adult sea cucumber coelom around the genital pore (Figure S3). However, a visibly weaker auto-fluorescent signal was also observed in the control samples.

### Comparison of the efficiency of *SRα* gene knockdown using lentivirus-mediated and dsRNA-based RNAi in intestines tissues

**Figure 4:**
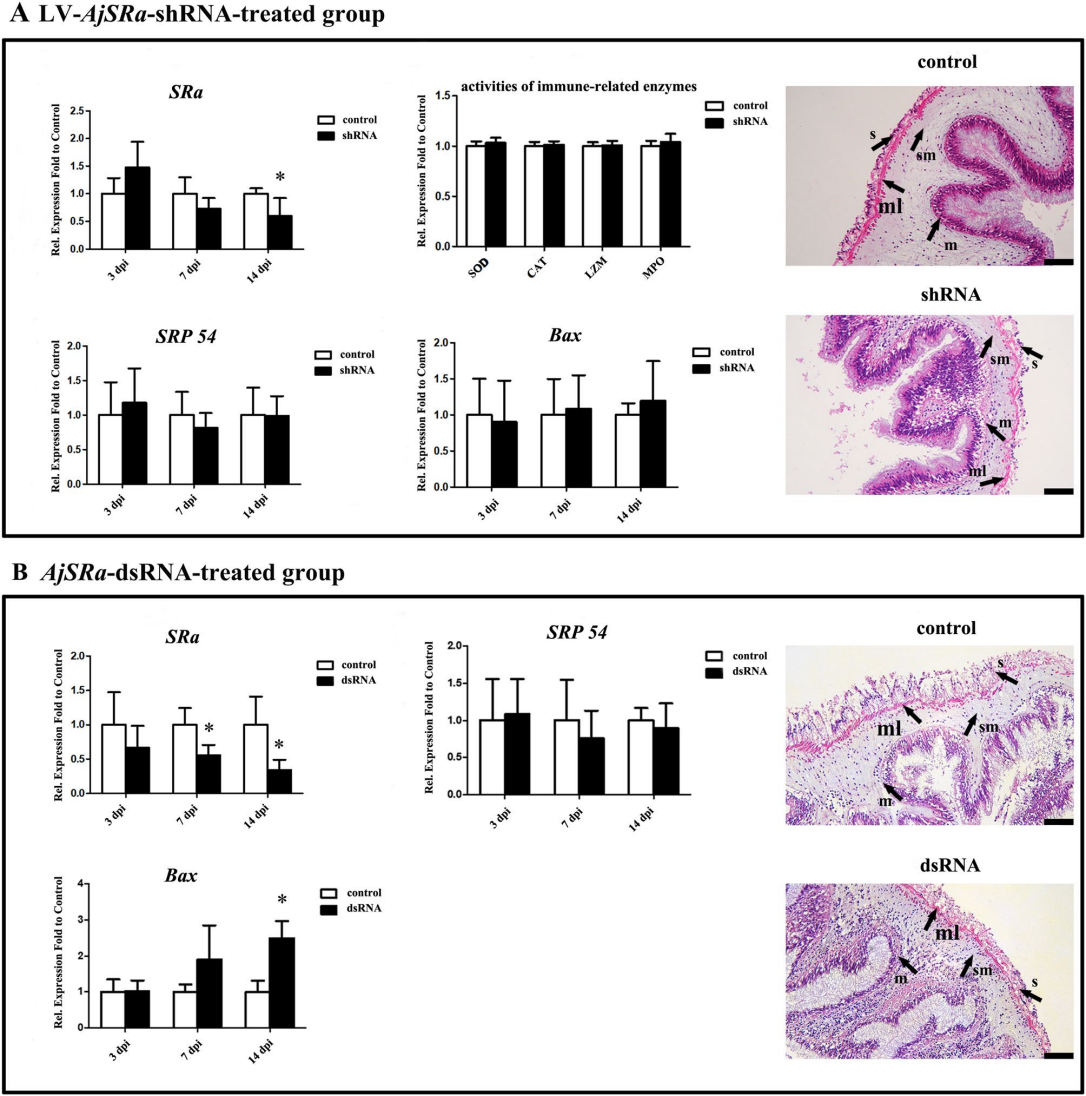
RNA interference (RNAi) of *SRα* in intestines. (**A**) RT-qPCR measured the mRNA expression level of *SRα*, *SRP54* and *Bax* at 3dpi, 7dpi and 14dpi after LV-*AjSRα*-shRNA treatment. Detection of immune-related enzyme activities and histological examination was performed at 14dpi after LV-*AjSRα*-shRNA treatment. (**B**) The mRNA expression level of *SRα*, *SRP54*, and *Bax* analysed and histological examination after *AjSRα*-dsRNA treatment. *NADH*, *ACTB*, and *TUBB* were normalized to a panel of reference genes between the LV-*AjSRα*-shRNA treated group and control group. *ACTB*, *RPS18* and *NDUFA13* were normalized to a panel of reference genes between the *AjSRα-*dsRNA treated group and control group. Each bar represents mean ± SD (n = 3). The asterisks indicate the significant differences between the knockdown group and the control group (*p* ≤ 0.05). dpi: days post-injection. s: serosa layer; sm: the inner connective tissue layer; ml: muscular layer; m: intestinal lumen epithelium. Bar = 50 μm.

Subsequently, we used different RNAi methods to generate loss-of-function mutants by injecting the virus of LV- *AjSRα*-shRNA or *AjSRα*-targeting dsRNA into adult sea cucumber coelom. As shown in Fig. 4A, the expression level of *SRα* did not show significant changes at 3 days post-injection (dpi) of LV-*AjSRα-*shRNA and 7 dpi. The *SRα* transcriptions dramatically decreased by 40.12% compared to control samples at 14 dpi. The activities of immune-related enzymes including superoxide dismutase (SOD), catalase (CAT), lysozyme (LZM) and myeloperoxidase (MPO) in intestines tissues show no significant change after the injection of LV-*AjSRα*-shRNA. Eukaryotic SRP composed of a 7S RNA and six different proteins (SRP9, SRP14, SRP19, SRP54, SRP68 and SRP72). Among these components, SRP54 is primarily responsible for binding to SRα delivering translational ribosomes to the ER membrane^8^. Therefore, we detected the mRNA expression level of *SRP54* after *SRα* knockdown. However, the transcription expression level of *SRP54* did not show significant changes in the knockdown group. According to previously reported findings, the upregulation of the *Bax* gene expression suggests the occurrence of apoptosis in the cells^28^. Nevertheless, in the lentivirus-mediated RNAi knockdown group at 14dpi, the transcripts of *Bax* exhibited no significant difference compared to the control group. Corresponding, there were no noticeable apoptosis cells or noticeable histological changes observed.

In dsRNA-based RNAi knockdown group, the transcripts of *SRα* show no significant change at 3dpi, but sharply downregulated to 44.45% to 65.82% at 7 dpi and 14 dpi (Fig. 4B). Knockdown of the *SRα* did not affect the expression levels of *SRP54*. In addition, the mRNA expression level of *Bax* were no significant change at 3dpi, but significantly increased to 1.89 and 2.49-fold at 7dpi and 14 dpi, respectively. However, no apoptotic cells and obviously histological change were found in intestines tissues after *AjSRα*-dsRNA treatment.

### Comparison of the efficiency of *SRα* gene knockdown using lentivirus-mediated and dsRNA-based RNAi in ovary tissues

**Figure 5:**
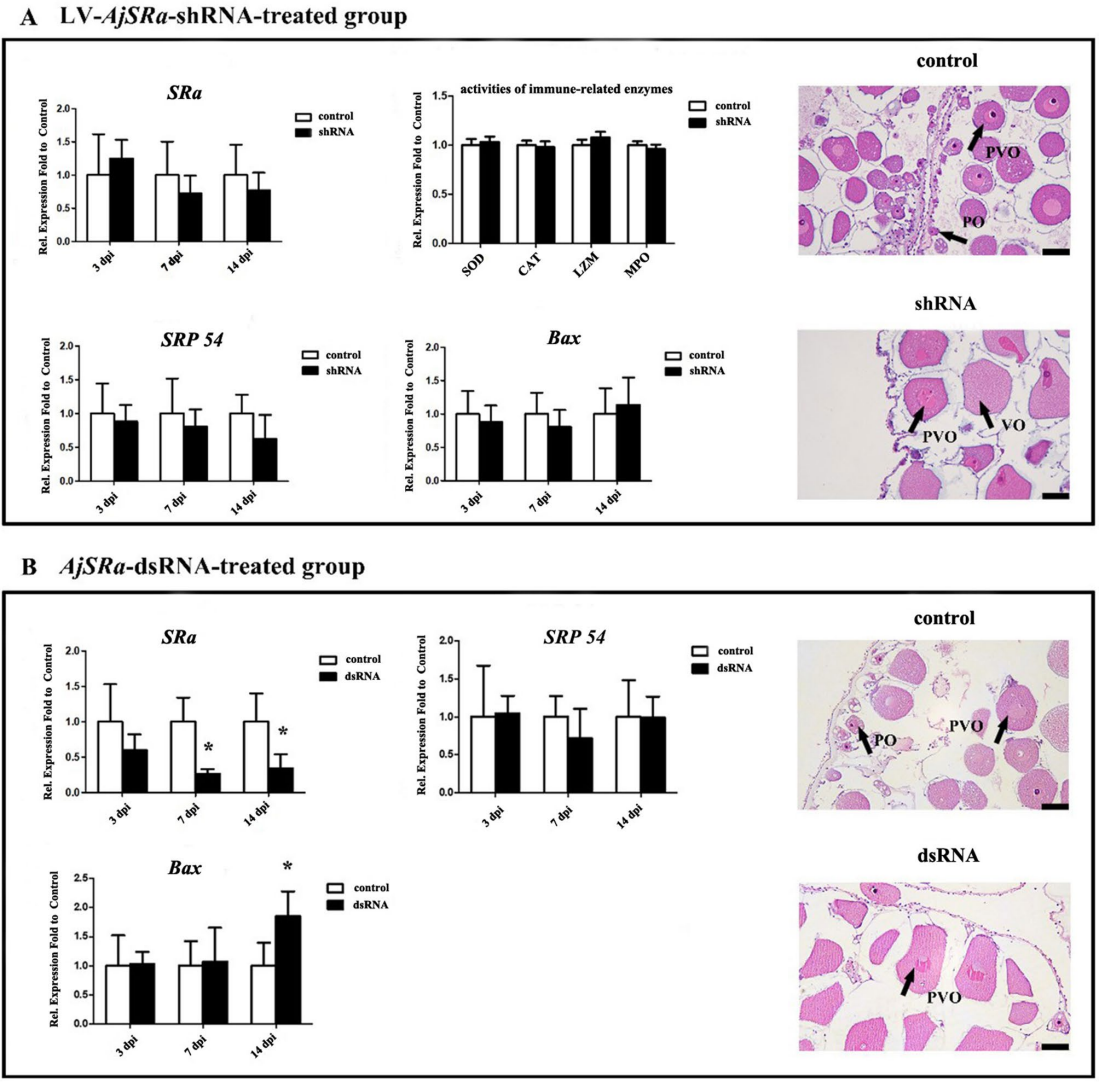
RNA interference (RNAi) of *SRα* in ovaries. (**A**) RT-qPCR measured the mRNA expression level of *SRα*, *SRP54* and *Bax* at 3dpi, 7dpi and 14dpi after LV-*AjSRα*-shRNA treatment. Detection of immune-related enzyme activities and histological examination was performed at 14dpi after LV-*AjSRα*-shRNA treatment. (**B**) The mRNA expression level of *SRα*, *SRP54*, and *Bax* analysed and histological examination after *AjSRα*-dsRNA treatment. *NADH*, *ACTB*, and *TUBB* were normalized to a panel of reference genes between the LV-*AjSRα*-shRNA treated group and control group. *ACTB*, *RPS18* and *NDUFA13* were normalized to a panel of reference genes between the *AjSRα-*dsRNA treated group and control group. Each bar represents mean ± SD (n = 3). The asterisks indicate the significant differences between knockdown group and the control group (*p* ≤ 0.05). dpi: days post-injection. PO: primary; PVO: previtellogenic oocytes; VO: vitellogenic oocytes. Bar = 50 μm.

In the ovaries of LV-*AjSRα-*shRNA-treated sea cucumbers, the *SRα* transcripts showed an overall decreasing trend. However, there is no significant variation in the data among the samples (Fig. 5A). Enzymes activities assay revealed that the SOD, LZM, CAT and MPO show no significant difference after the injection of LV- *AjSRα*-shRNA virus compared with controls. The transcripts of *SRP54* and *Bax* were not show significantly suppressed compared to the control group (Fig. 5A).

Meanwhile, the expression of *SRα* mRNA were significantly decreased by 39.95%, 73.40% and 66.00% at 3 dpi, 7 dpi and 14 dpi in the ovaries of *AjSRα*-dsRNA treated sea cucumbers (Fig. 5B). The expression of *SRP54* were also not significantly affected by depletion of *SRα*. The transcripts of *Bax* were no significant change at 3dpi and 7dpi but increased by 84.94% at 14 dpi after *AjSRα*-dsRNA treatment. No apoptotic cells and obviously histological change were detected both in LV-*AjSRα*-shRNA treated and *AjSRα*-dsRNA treated ovaries (Fig. 5).

## Discussion

The SRP receptor is necessary for targeting nascent secretory proteins to the endoplasmic reticulum membrane in mammalian cell^4,29^. *SR*a is an ancient gene composed of a conserved NG domain (SRP54_N domain and SRP54 domain) and plays an important role in protein translocation^30^. Generally, the *SRa* transcripts and proteins are ubiquitously expressed in both eukaryotic and prokaryotic cells, and they can serve as useful markers for the outer ER membrane^28,31^. The *A. japonicus* homologue of SRa contains three conserved domains, including the SRP-alpha N domain, SRP54_N domain and SRP54 domain. Among them, the SRP54_N domain and SRP54 domain is responsible for GTP binding. Consistent with findings in other species, *AjSRa* transcripts can be detected in all analysed tissues and throughout all developmental stages of embryogenesis indicating its significant role in protein targeting pathway.

Lack of effective tools has become a bottleneck for studying gene function in non-model organisms. RNA interference can significantly reduce the expression of target genes both in vivo and in vitro, and it is widely employed for loss-of-function studies in aquaculture animals^23,32–34^. RNAi can be induced in organicism using siRNAs, dsRNAs and shRNAs^35–37^. Using lentiviral to deliver shRNA to induce RNAi expression or for delivery Cas9 and gRNA to achieve genome editing has been widely used in vertebrates, especially in mamanis^26,38–40^. Generally, lentiviral vectors do not elicit any effective cellular immune response from the host^41^, and transgene expression mediated by lentiviral vectors can persist for several months^42^. However, targeting the PAI-2 mRNA with vector-derived shRNA induced a rapid cytotoxic effect in human tumor cell lines, and failed to maintain stable gene silencing in long-term cell culture^43^. We first attempt to use lentiviral vectors for delivering shRNA in invertebrates, and revealed that lentiviral vectors do not induce an upregulation of a panel of immune-related enzymes.

Combining the results of *SRα* gene down-regulation and the fluorescence images of infected tissues, it seems that lentiviral-mediated transgenesis may work in echinoderms. However, the auto-fluorescent signal in control images cannot be overlooked. Typically, lentivirus transduction relies on the specific recognition of the CR2/3 regions of the mammalian LDLR by the VSV-G envelope protein^44,45^. A mammalian homologous LDLR gene has been annotated in the genome of *A. japonicus*, and the identity between the sea cucumber and human CR2 domain and CR3 domain is 45.00% and 52.78%, respectively (Figure S4). In the future, it is worthwhile to explore the interaction between the LDLR of *A. japonicus* and the VSV-G envelope protein, as well as the detection of proviral integration of lentivirus in the *A. japonicus* genome using a sequencing-based method.

In previous studies, we directly injected dsRNAs targeting several sex-related genes into the coelom around the genital pore of sea cucumbers to achieve gene silencing^21–23^. In the present study, the lentivirus-mediated interference was inferior to that achieved with dsRNA injection alone in sea cucumbers. Consequently, we chose to pursue the dsRNA method in lieu of evaluating the functionality of the lentiviral transductions. The upregulation of *Bax* gene expression suggests the possibility of apoptosis occurring in the cells^28,46^. In humans, the variants of *SRα* have a pathogenic, and the vitro experiments show that the mutation of *SRα* increases the susceptibility to apoptosis^10^. Therefore, we speculate that although the histological morphology remains unchanged in intestines and ovaries, the up-regulation of *Bax* implies that apoptosis may occur in future. In the Chinese mitten crab (*Eriocheir sinensis*), injecting long *dmrt*-like dsRNA for one month led to the atrophy of seminiferous tubules and fewer male germ cells in the seminiferous tubules^47^. In the prawn (*Macrobrachium rosenbergii*), silencing the insulin-like androgenic gland hormone gene (IAGs) through a long-term in vivo injection of long dsRNAs resulted in complete and functional sex reversal of male freshwater prawns^34^. In future dsRNA-based RNAi knockdown experiments, increasing the number of injections and extending the duration of the injection experiment should be taken into full consideration.

## Materials and methods

### Sample preparation and genetic sex identification

Adult sea cucumbers were purchased from Dalian Yongjia Group Corporation (39.70'N, 121.50'E). The genetic sex identified was performed by PCR amplification reaction according to our previous report^48^. Sea cucumber embryos and larvae were collected from the Key Laboratory of Mariculture Stock Enhancement in the North China’s Sea, Ministry of Agriculture and Rural Affairs, Dalian Ocean University. This study didn't involve any endangered or protected species.

### Sequence clone and analysis

According to the *A.japonicus* genome sequences (taxid: 307972), the full-length cDNA sequences of *AjSRα* have been obtained by 5’ and 3’ rapid amplification of cDNA ends (RACE). The primers used in this study were designed on the software of Primer Premier 5 (Table S1)^49,50^. The amino acid sequence of *AjSRα* deduced and conserved structural domains predicted were performed with open reading frame (ORF) Finder and SMART program. The multiple amino acid sequence alignments were using BioEdit software. The phylogenetic tree was constructed by the maximum likelihood method in MEGA-7 software, with 1000 replicates of bootstrap analysis.

### RNA extraction and real-time quantitative PCR analysis

Total RNA was extracted from different tissues in adult sea cucumbers, including the longitudinal is muscle, ovaries, testes, intestines, stomach, respiratory tree, coelomocytes, and tube feet, using TRIzol™ reagent (Invitrogen, USA). Meanwhile, the stages of early development, including the embryonic and larvae, were also collected to extract total RNA. RNAs concentration were measured by NanoDrop 2000, and RNAs quality were verified by using 1% agarose gel. Total RNAs (1 µg) were pooled for first-strand cDNA synthesis.

All RT-qPCR amplification reactions were mixtures, including 10 µL of RT-qPCR SYBR Green Master Mix (Yeasen, Shanghai, China), 2 µL of cDNA, 0.8 µL (10 µM) of each primer, and 6.4 µL of RNase-free water. The RT-qPCR program was as follows: denaturation at 95 °C for 10 min; then, 40 cycles of 95 °C for 15 s, 60 °C for 1 min. According to the previous report, housekeeping genes exhibit different expression levels under various conditions^51^. To select stable reference genes, we analysed the expression patterns of several housekeeping genes, including *Actin Beta* (*ACTB*), 40S ribosomal protein S18 (RPS18), *NADH dehydrogenase* (*NADH*), *NADH:Ubiquinone Oxidoreductase Subunit A13* (*NDUFA13*), *Tubulin Alpha* (*TUBA*) and *Tubulin Beta Class I* (*TUBB*) using geNorm analysis^52^. The results showed that *NADH*, *ACTB*, and *TUBB* were stable reference genes for normalization between the lentiviral treatment tissues and control samples (Table S2), and *ACTB*, *RPS18* and *NDUFA13* proved to be stable for normalization between the dsRNA treatment tissues and control samples (Table S3). The dynamic change in expression levels of the analysed genes between the LV-*AjSRα*-shRNA treatment tissues and control samples were normalized using the geometric mean of *NADH*, *ACTB*, and *TUBB*, and determined using the 2^−ΔΔCt^ method. Meanwhile, *ACTB*, *RPS18* and *NDUFA13* were used as a panel of reference genes to detect the change in genes expression levels between the *AjSRα*-dsRNA treatment tissues and control samples. All data were obtained from three independent biological replicates.

For statistical analysis, one-way ANOVA was utilized to evaluate significant differences in *SRα* mRNA distribution in different tissues and embryogenesis. Bonferroni corrections were used to adjust probability (p) values, *p* ≤ 0.0017 were considered statistically significant. Student’s t test was utilized to evaluate significant difference between the RNA interference group and control group. Probability values (*p*) ≤ 0.05 were considered statistically significant.

### Lentiviral *AjSRα*-shRNA construction and generation

The construction of the lentiviral vector and the generation of the lentivirus were completed by GenePharma Company (Shanghai, China). The shRNA was designed based on the *AjSRα* mRNA sequence and the sequence is as follow: *AjSRα*-shRNA: 5’-GCCAAGAACGTTGCACAAGAA-3’. The 21 nt *AjSRα*-shRNA sequence, a 9 nt loop sequence (5’-TTCAAGAGA-3’), and the antisense strand of *AjSRα*-shRNA sequence were inserted into the plasmid LV3 (pGLVH1/GFP-Puro) (GenePhrama, Shanghai, China) between the *BamHI* and *EcoRI s*ites in sequence (LV-*AjSRα*-shRNA). A poly-T terminator was positioned before the *EcoRI* site, and the sequencing results of the inserted sequence can be found in the Figure S5. Meanwhile, nonsense shRNA (5'-TTCTCCGAACGTGTCACGT-3') was cloned into the plasmid LV3 as a negative control (LV-NC-shRNA) using the same method.

To generate lentivirus, HEK293T cells were cultured in Dulbecco's Modified Eagle's medium (DMEM) with 10% fetal bovine serum (FBS) on 10 cm plates at 37 °C with 5% CO_2_. Once they reached 80–90% confluence, the HEK293T cells were transferred to 15 cm plates and cultured overnight. For co-transfection, three packaging plasmids (pGag/Pol, pRev, and pVSV-G) and lentiviral vector *AjSRα*-shRNA (LV-*AjSRα*-shRNA) or the control (LV-NC-shRNA) were mixed with 300 µL of RNAi-Mate transfection reagent (GenePharma, Shanghai, China) and incubated for 25 min at room temperature. Subsequently, the mixture was added to HEK293T cells and incubated in 8 mL of DMEM at 37 °C with 5% CO_2_ for 4–6 h. Following this, the transfection medium was removed and replaced with DMEM culture medium containing 10% FBS (without antibiotics). At 72 h post-transfection, collect the incubated supernatant and centrifuge for 4 min at 4 °C and 4000 rpm, filter it through a 0.45 µm filter, followed by ultracentrifugation for 2 h at 4 °C and 20,000 rpm. Then, collect the concentrated solution and store it at −80 °C. To evaluation virus titer, the lentivirus stock solution was diluted into four gradients: 10^–1^, 10^–^^2^, 10^–^^3^ and 10^–^^4^ using DMEM culture medium with 10% FBS. Then, 100 µL of viruses at different concentrations were added into the HEK293T cells in a 96-well plate at a concentration of 3 × 10^4^ cells/well and culture for 24 h. After replacing the culture medium with no virus, HEK293T cells continued to culture for 72 h. Count the proportion of GFP expression in transfected cells and calculate the virus titer using the following formula:

$${\mathrm{TU}}/\mu {\mathrm{L}} = \left( {{\mathrm{P}} \times {\mathrm{N}}/{1}00 \times {\mathrm{V}}} \right) \times {1}/{\mathrm{DF}}$$

TU is the transducing units, P is the proportion of cells expressing GFP fluorescence, N is the number of cells at the time of transfection, V is the volume of virus dilution added per well, DF is the dilution factor. Eventually, a viral titer of 1 × 10^9^ TU/ml was obtained.

### RNAi by LV-*AjSRα*-shRNA

A total of 18 healthy female sea cucumbers weighing 90 ± 15 g was collected and randomly assigned into two groups: LV-*AjSRα*-shRNA knockdown group (n = 9) and LV-NC-shRNA group (n = 9). The knockdown group was injected with high titer virus of LV-*AjSRα*-shRNA. Before injection, 100ul virus of the LV-*AjSRα*-shRNA stock solution with a titer of 1*10^9^ TU/ml was aspirated and mixed with 200ul PBS. Then, approximately 100 ul of the mixture injected per 50 g body weight. The control group was injected with the same dosages of the disordered control virus of LV-NC-shRNA. Injections were administered every two days. Samples were performed on days 3, 7, and 14, respectively, and three sea cucumbers from each group were collected for further analysis. The intestines and gonads were surgically sampled for RT-qPCR, histological and immunofluorescence analysis.

### RNAi by dsRNA

Three dsRNAs targeting *AjSRα* mRNA were designed by an online software (https://www.dkfz.de/signaling/e-rnai3/) and synthesized by the T7 RiboMAXTM Express RNAi System according to the instruction of manufacture (Promega). Subsequently, a total of 18 healthy female sea cucumbers (weight, 90 ± 15 g) were used to be randomized into the *AjSRα*-dsRNA group (n = 9) and control group (n = 9). The three kinds of *AjSRα*-specific dsRNA were mixed and injected into the knockdown group at approximately 100 ug per 50 g of body weight. Meanwhile, the same concentration of GFP dsRNA was injected into the control group. Injections were administered every two days. Samples were performed on days 3, 7, and 14, respectively, and three sea cucumbers from each group were collected for further analysis. The intestines and gonads were surgically sampled for RT-qPCR and histological analysis.

### Immunofluorescence localization

To detect the expression of GFP protein location, immunofluorescence was performed. The tissues of intestines and ovaries were fixed in 4% paraformaldehyde (PFA) at 4°C overnight. After balanced in 30% sugar solution, the samples were embedded in optimal cutting temperature compound (OCT) compound and stored at − 80 ◦C until use. Serial sections were 6-µm thick on poly-L-lysine-coated glass slides with frozen slicer (LeicaCM 1950). Then the tissues were rehydrated with PBS, and blocked with 5% non-fat powdered milk and 0.5% Triton X-100 for 1 h at room temperature. Subsequently, sections were incubation with GFP antibody (1:500) at 4°C overnight. Eventually, signal detection was performed using Leica microscopes (Lecia DM4B).

### Histology examination

The intestines and ovaries tissues were obtained and fixed with 4% PFA in phosphate buffer saline (PBS) overnight at 4°C and embedded in paraffin. For histochemical analyses, 6-μm-thick sections were cut and deparaffinized with xylene, rehydrated with ethanol. After that, sections were stained with hematoxylin and differentiation with hematoxylin differentiation solution. Subsequently, the sections were stained with eosin, dehydration by absolute ethanol, and cleaned with xylene. Finally, the neutral gum was used to sealing the sections. Digital images were collected on the microscope (Leica DM4B).

## Supplementary Information

Supplementary Information.

## Data Availability

The dataset used and analysed during the current study is available from the corresponding author on reasonable request.
