# Supplementary material for: A signal recognition particle receptor gene from the sea cucumber, Apostichopus japonicus
Source: Sci Rep. 2023 Dec 27;13:22973. doi: 10.1038/s41598-023-50320-z (PMC10752883; doi:10.1038/s41598-023-50320-z)
Supplement: Supplementary file 1 — Supplementary Information. [file 41598_2023_50320_MOESM1_ESM.doc]

**A signal recognition particle receptor gene from the sea cucumber, *Apostichopus japonicus***

**Jian Zhang^1,2^, Zhihui Sun^2*^, Weiyi Su^2^, Zengdong Wang^3^, Weihan Meng^2^, Yaqing Chang^1,2*^**

1. School of Life Science, Liaoning Normal University, Dalian, 116029, China

2. Key Laboratory of Mariculture& Stock Enhancement in North China Sea, Ministry of Agriculture and Rural Affairs, Dalian Ocean University, Dalian, 116023, China

3. Shandong Anyuan Aquaculture Co. Ltd, Yantai, 264000, China

* Corresponding Author：

Yaqing Chang ([changlab@hotmail.com](mailto:changlab@hotmail.com)); Zhihui Sun ([sunzhihui@dlou.edu.cn](mailto:sunzhihui@dlou.edu.cn).)

Tel.: +86-411-84762691 (Yaqing Chang); +86-0411-84762695 (Zhihui Sun).

**Figure S1**: Amino acid alignments of *Aj*SRα with other homologues. The black frame indicates the SRP-alpha N domain, SRP54 N domain and SRP54 domain, respectively.


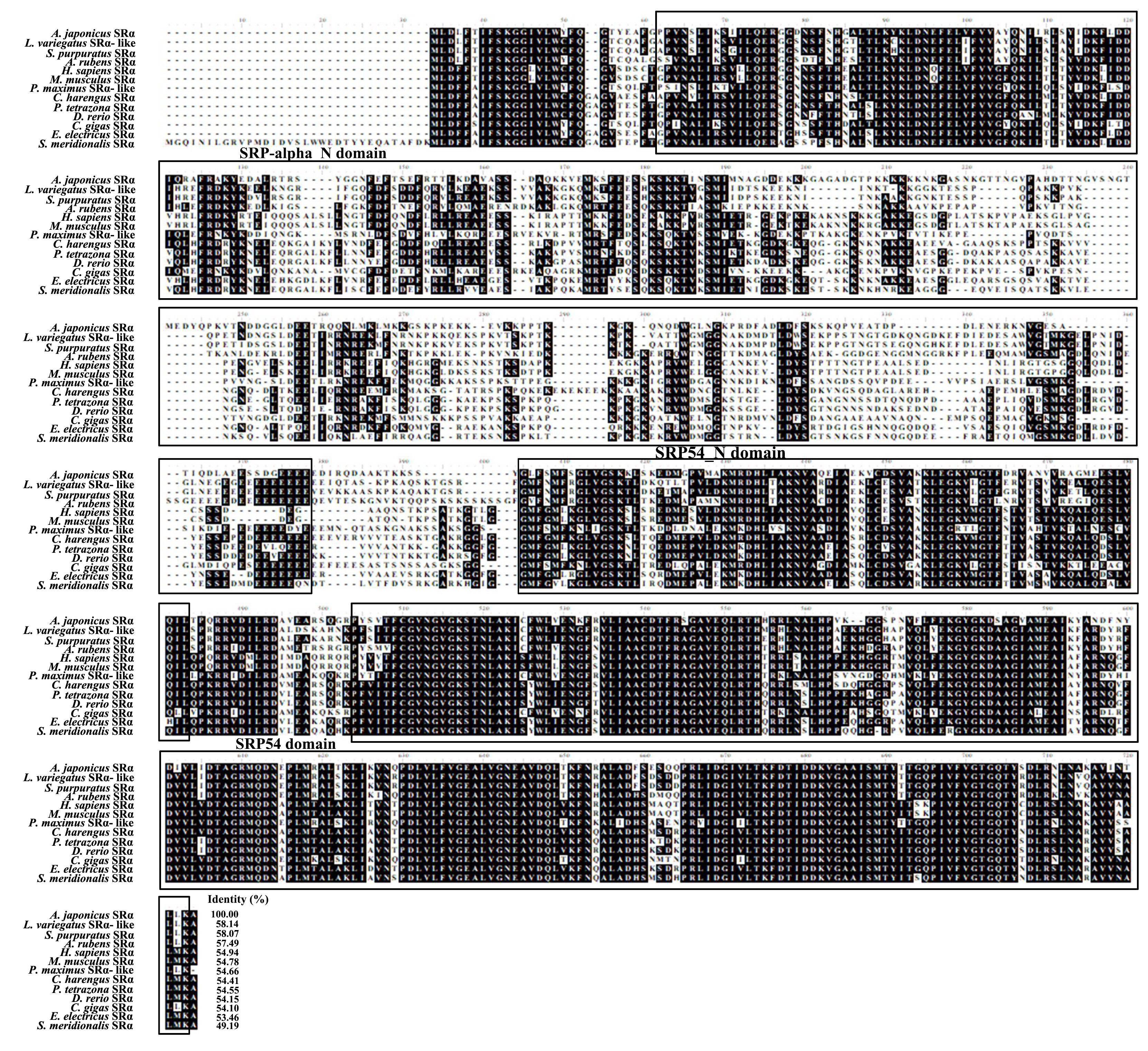


**Figure S2:** Conserved domains alignment of *Aj*SRα with other homologues. (A) SRP-alpha N domain alignment of *Aj*SRα with other homologues. (B) SRP54 N domain alignment of *Aj*SRα with other homologues. (C) SRP54 domain alignment of *Aj*SRα with other homologues.


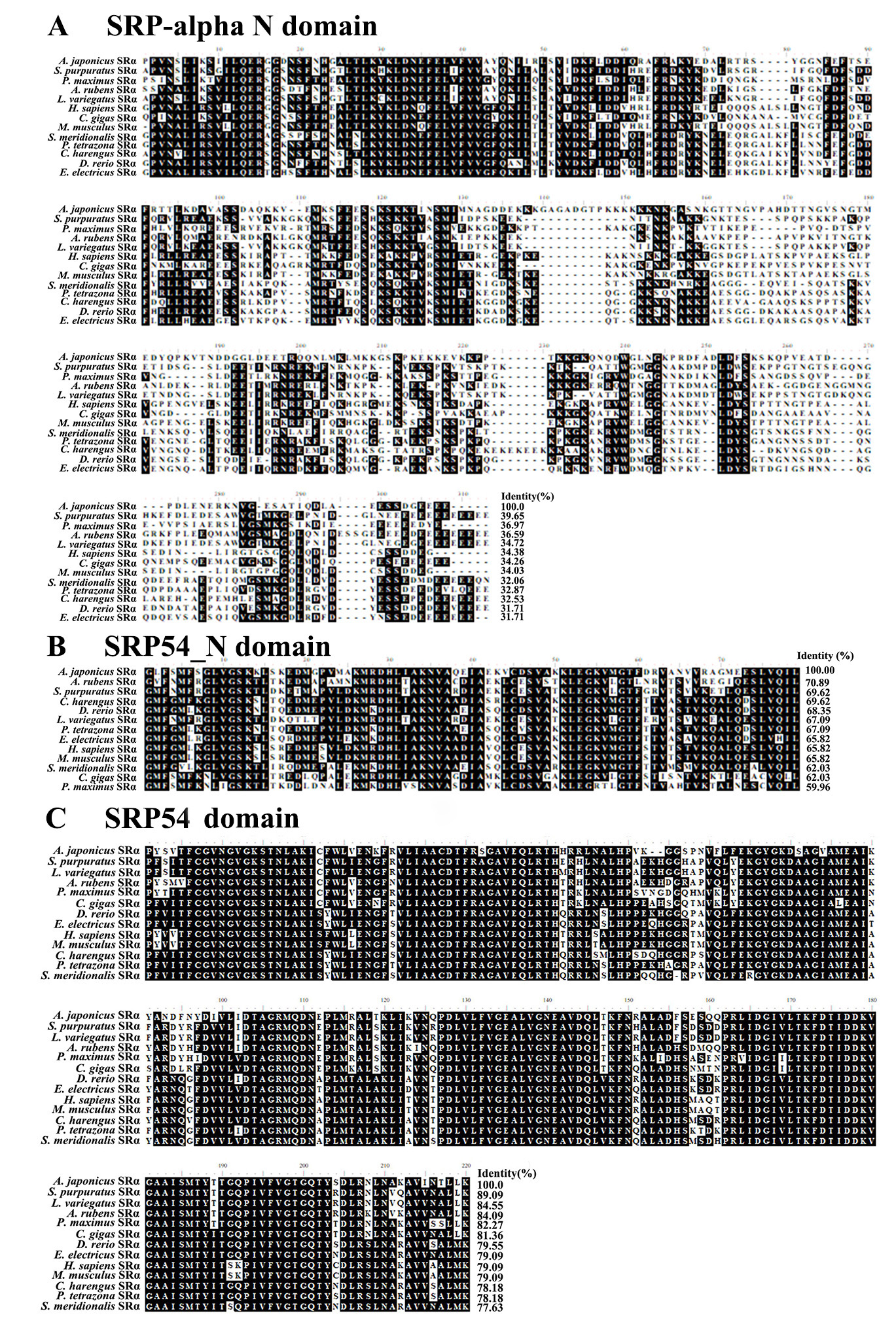


**Figure S3.** GFP expression after the infection of LV- *AjSRα*-shRNA in intestines and ovary tissues of adult sea cucumbers. s: serosa layer; sm: the inner connective tissue layer or the submucosa; PVO: previtellogenic oocytes. Bar = 50 μm.


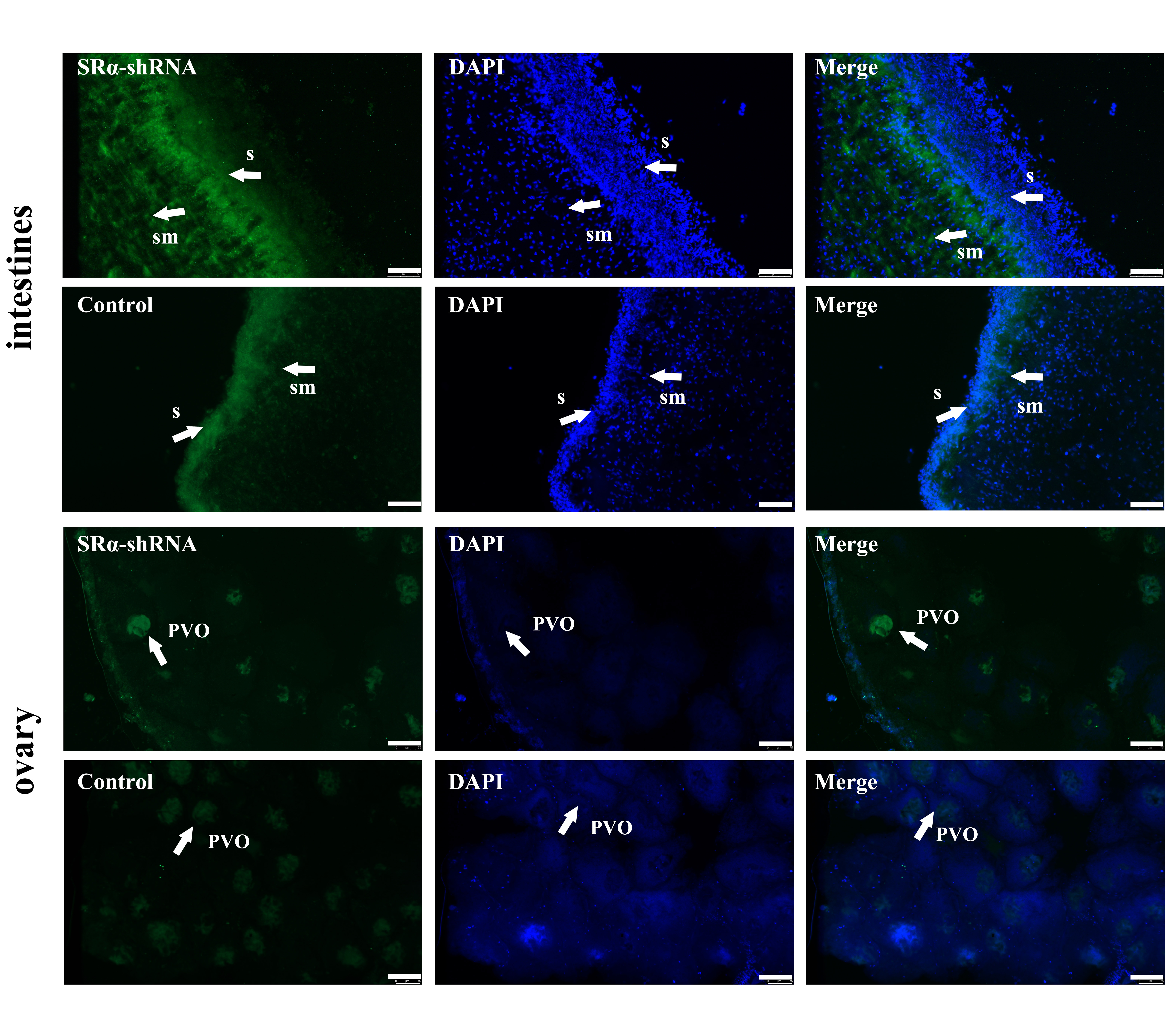


**Figure S4**: CR2 domain and CR3 domain of LDLR alignment of sea cucumber (*A. japonicus*) with human (*H. sapiens*). (A) CR2 domain of LDLR alignment of sea cucumber (*A. japonicus*) with human (*H. sapiens*). (B) CR3 domain of LDLR alignment of sea cucumber (*A. japonicus*) with human (*H. sapiens*). The 6 cysteines were marked with asterisks (*), and the four acidic residues were marked with I, II, III, IV.


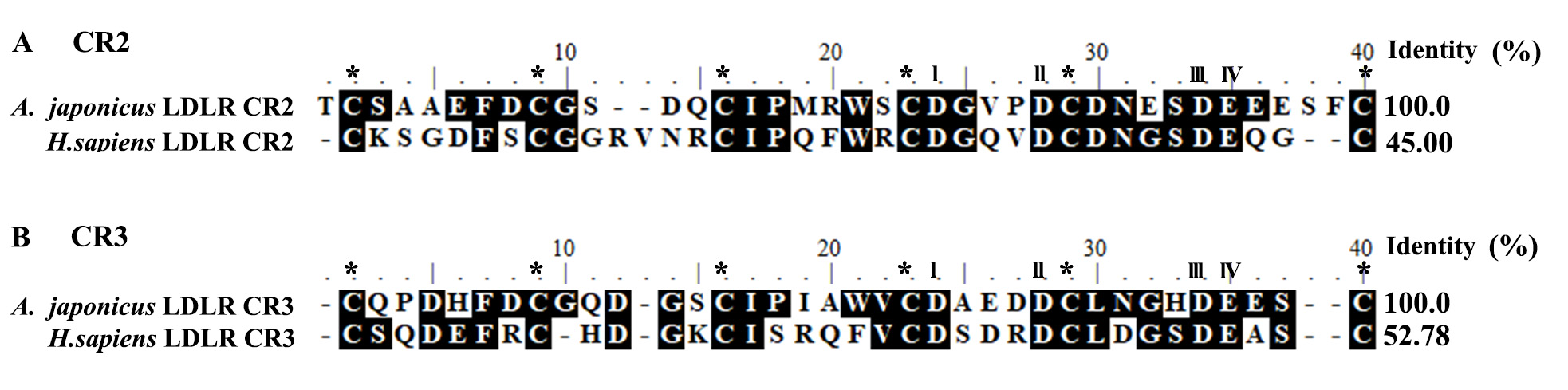


**Figure S5:** Insert *AjSRα*-shRNA sequence in LV3 (pGLVH1/GFP-Puro) plasmid.


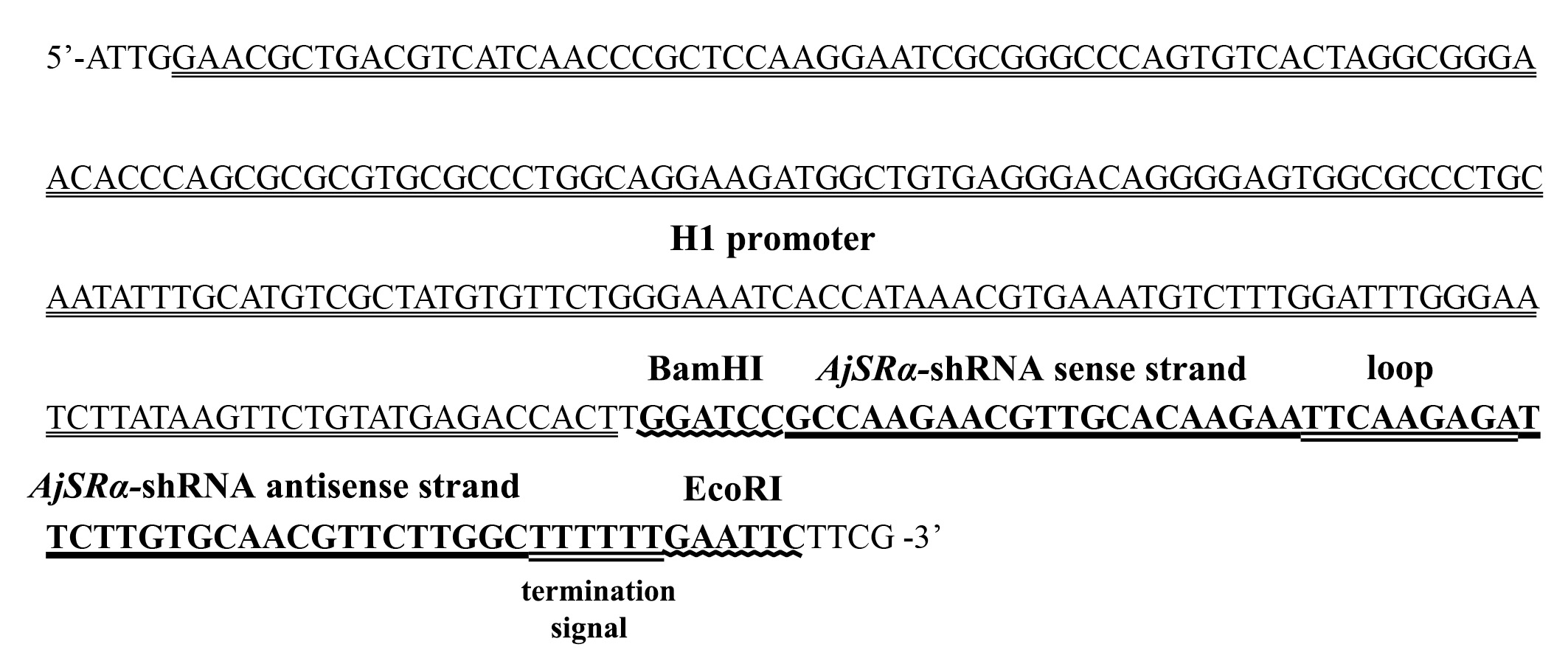


| Primer name | Purpose | Sequence (5′ to 3′) |
| --- | --- | --- |
| *SRα-5’-1* | RACE | ACCAGTTCAAATTCAT |
| *SRα-5’-2* | RACE | TCCATGGTTAAAAGAGTTGTCT |
| *SRα-5’-3* | RACE | ATAAGTGAGTTGACGGGTGG |
| *SRα-3’-1* | RACE | CTGTGATTCAGTTGCCAAGAAACTGG |
| *SRα-3’-2* | RACE | TAATGGGAACTTTTGACCGTGTGG |
| *UPM-1* | RACE | TAATACGACTCACTATAGGGCAAGCAGTGGTATCAACGCAGAGT |
| *UPM-2* | RACE | CTAATACGACTCACTATAGGGC |
| *SRα-F* | RT-qPCR | GGGAAGGATTCAGCTGGAGTCGC |
| *SRα-R* | RT-qPCR | GGCTGCTCCGACCTTATCATCGAT |
| *SRα-ds1-F* | dsRNA | **TAATACGACTCACTATAGGG**AGGGTGGAATTGTTCTGTG |
| *SRα-ds1-R* | dsRNA | **TAATACGACTCACTATAGGG**GTAGTCTTCCATGGTGCCGT |
| *SRα-ds2-F* | dsRNA | **TAATACGACTCACTATAGGG**ACGGCACCATGGAAGACTAC |
| *SRα-ds2-R* | dsRNA | **TAATACGACTCACTATAGGG**CACGGTCAAAAGTTCCCATT |
| *SRα-ds3-F* | dsRNA | **TAATACGACTCACTATAGGG**GGATGGAGGAGTCGTTGGTA |
| *SRα-ds3-R* | dsRNA | **TAATACGACTCACTATAGGG**CAGGTCGGGTTGATTCACTT |
| *SRP54-F* | RT-qPCR | GCATCCATAGGACAAGCATGCGAAT |
| *SRP54-R* | RT-qPCR | GAATGGGCCCATCTTCATAATGTTC |
| *Bax-F* | RT-qPCR | CGTGTAGTTGGGGTAGGGTGGTAAT |
| *Bax-R* | RT-qPCR | CTTGTCCCTCCAGTCATTGGCTTTG |
| *NADH-F* | RT-qPCR | GTCCTACGACCCAATCTGGA |
| *NADH-R* | RT-qPCR | ATGAGCCTTGGTTACGTTGG |
| *ACTB-F* | RT-qPCR | AAGGTTATGCTCTTCCTCACGCT |
| *ACTB-R* | RT-qPCR | GATGTCACGGACGATTTCACG |
| *TUBB-F* | RT-qPCR | GAAAGCCTTACGACGGAACA |
| *TUBB-R* | RT-qPCR | CACCACGTGGACTCAAAATG |
| *RPS18 -F* | RT-qPCR | GACGGCAAGTTCAACCAGAT |
| *RPS18 -R* | RT-qPCR | GTGCTGACCTCTGACACGAA |
| *NDUFA13-F* | RT-qPCR | GACATTCTGAAGCAACTGCAAC |
| *NDUFA13-R* | RT-qPCR | AACATTGCCTCATTCTGTTCCT |
| *TUBA-F* | RT-qPCR | TGGAAGATGAGGAACCCTTG |
| *TUBA-R* | RT-qPCR | GGAAAGGAGCAAATCGATCA |

Table S1. Sequences of the primers used for PCR. The bold font indicates T7 primer.

TableS2 Stable reference genes selected in LV-*AjSRα*-shRNA group and LV-NC-shRNA group by geNorm analysis

| **Sample name** | **ACTB(2^-∆Ct^)** | **NADH(2^-∆Ct^)** | **TUBB(2^-∆Ct^)** | **Normalization Factor** |
| --- | --- | --- | --- | --- |
| Intestine-LV-AjSRα-shRNA 14dpi-A | 1 | 0.057114466 | 0.429282718 | 1.625 |
| Intestine-LV-AjSRα-shRNA 14dpi-B | 1 | 0.036397925 | 0.205897754 | 1.0947 |
| Intestine-LV-AjSRα-shRNA 14dpi-C | 1 | 0.022718321 | 0.189464571 | 0.91 |
| Intestine-LV-NC-shRNA 14dpi-A | 1 | 0.037681495 | 0.179244406 | 1.0574 |
| Intestine-LV-NC-shRNA 14dpi-B | 1 | 0.036906021 | 0.082469244 | 0.8107 |
| Intestine-LV-NC-shRNA 14dpi-C | 1 | 0.047366143 | 0.216134308 | 1.2147 |
| Ovary-LV-AjSRα-shRNA 14dpi-A | 1 | 0.024860515 | 0.25348987 | 1.0333 |
| Ovary-LV-AjSRα-shRNA 14dpi-B | 1 | 0.068393356 | 0.236514412 | 1.4148 |
| Ovary-LV-AjSRα-shRNA 14dpi-C | 1 | 0.022561394 | 0.03794359 | 0.5312 |
| Ovary-LV-NC-shRNA 14dpi-A | 1 | 0.011438169 | 0.213158723 | 0.7529 |
| Ovary-LV-NC-shRNA 14dpi-B | 1 | 0.038473263 | 0.406126198 | 1.3985 |
| Ovary-LV-NC-shRNA 14dpi-C | 1 | 0.03794359 | 0.712025098 | 1.6786 |
| Intestine-LV-AjSRα-shRNA 7dpi-A | 1 | 0.022250784 | 0.22221067 | 0.9530 |
| Intestine-LV-AjSRα-shRNA 7dpi-B | 1 | 0.033261568 | 0.325335464 | 1.2373 |
| Intestine-LV-AjSRα-shRNA 7dpi-C | 1 | 0.025916236 | 0.22221067 | 1.0027 |
| Intestine-LV-NC-shRNA 7dpi-A | 1 | 0.043284671 | 0.325335464 | 1.3509 |
| Intestine-LV-NC-shRNA 7dpi-B | 1 | 0.01674646 | 0.085971364 | 0.6317 |
| Intestine-LV-NC-shRNA 7dpi-C | 1 | 0.047366143 | 0.207329886 | 1.1979 |
| Ovary-LV-AjSRα-shRNA 7dpi-A | 1 | 0.029157281 | 0.114228931 | 0.8534 |
| Ovary-LV-AjSRα-shRNA 7dpi-B | 1 | 0.01067219 | 0.438302861 | 0.9356 |
| Ovary-LV-AjSRα-shRNA 7dpi-C | 1 | 0.036651092 | 0.141610486 | 0.9685 |
| Ovary-LV-NC-shRNA 7dpi-A | 1 | 0.020333466 | 0.161544104 | 0.8316 |
| Ovary-LV-NC-shRNA 7dpi-B | 1 | 0.025033434 | 0.269807059 | 1.0574 |
| Ovary-LV-NC-shRNA 7dpi-C | 1 | 0.013792234 | 0.168404197 | 0.7408 |
| Intestine-LV-AjSRα-shRNA 3dpi-A | 1 | 0.031467361 | 0.072795849 | 0.7374 |
| Intestine-LV-AjSRα-shRNA 3dpi-B | 1 | 0.048697786 | 0.143587294 | 1.0697 |
| Intestine-LV-AjSRα-shRNA 3dpi-C | 1 | 0.028164077 | 0.166085727 | 0.9356 |
| Intestine-LV-NC-shRNA 3dpi-A | 1 | 0.051118879 | 0.20166044 | 1.2175 |
| Intestine-LV-NC-shRNA 3dpi-B | 1 | 0.032352029 | 0.203063099 | 1.0477 |
| Intestine-LV-NC-shRNA 3dpi-C | 1 | 0.035648866 | 0.197510328 | 1.0722 |
| Ovary-LV-AjSRα-shRNA 3dpi-A | 1 | 0.032352029 | 0.578344092 | 1.4851 |
| Ovary-LV-AjSRα-shRNA 3dpi-B | 1 | 0.012430258 | 0.351111219 | 0.9142 |
| Ovary-LV-AjSRα-shRNA 3dpi-C | 1 | 0.013888167 | 0.244855074 | 0.8412 |
| Ovary-LV-NC-shRNA 3dpi-A | 1 | 0.019640834 | 0.188155843 | 0.8649 |
| Ovary-LV-NC-shRNA 3dpi-B | 1 | 0.01286861 | 0.2381595 | 0.8126 |
| Ovary-LV-NC-shRNA 3dpi-C | 1 | 0.013888167 | 0.295248165 | 0.8954 |
|  |  |  |  |  |
| **M<1.5** | **0.77** | **0.894** | **0.953** |  |

TableS3 Stable reference genes selected in *AjSRα*-dsRNA group and *GFP*-dsRNA group by geNorm analysis

| **Sample name** | **ACTB(2^-∆Ct^)** | **TUBB(2^-∆Ct^)** | **NADH(2^-∆Ct^)** | **RPS18(2^-∆Ct^)** | **TUBA(2^-∆Ct^)** | **NDUF(2^-∆Ct^)** | **Normalization Factor** |
| --- | --- | --- | --- | --- | --- | --- | --- |
| Intestine-*AjSRα*-dsRNA 14dpi-A | 1 | 0.036146506 | 0.10153155 | 0.806641759 | 0.062068281 | 0.032128557 | 1.7164 |
| Intestine-*AjSRα*-dsRNA 14dpi-B | 1 | 0.027204705 | 0.106579361 | 0.678302164 | 0.072293011 | 0.04972103 | 1.9045 |
| Intestine-*AjSRα*-dsRNA 14dpi-C | 1 | 0.083620472 | 0.171942727 | 1.125058485 | 0.125869444 | 0.080214119 | 2.798 |
| Intestine-GFP-dsRNA 14dpi-A | 1 | 0.01991501 | 0.169575541 | 0.329876978 | 0.031686234 | 0.013139006 | 0.9278 |
| Intestine-GFP-dsRNA 14dpi-B | 1 | 0.024860515 | 0.071793647 | 0.368567304 | 0.047366143 | 0.027584469 | 1.2696 |
| Intestine-GFP-dsRNA 14dpi-C | 1 | 0.038207509 | 0.052192995 | 0.687770909 | 0.056328154 | 0.035896824 | 1.6551 |
| Ovary-*AjSRα*-dsRNA 14dpi-A | 1 | 0.006524124 | 0.010598471 | 0.105112052 | 0.013792234 | 0.012259127 | 0.5565 |
| Ovary-*AjSRα*-dsRNA 14dpi-B | 1 | 0.016630784 | 0.176776695 | 0.888842681 | 0.016401823 | 0.014377932 | 1.0312 |
| Ovary-*AjSRα*-dsRNA 14dpi-C | 1 | 0.019505165 | 0.133971683 | 1.790050142 | 0.042985682 | 0.073302184 | 2.3487 |
| Ovary-GFP-dsRNA 14dpi-A | 1 | 0.013696964 | 0.2381595 | 0.343885455 | 0.017948412 | 0.005448217 | 0.6526 |
| Ovary-GFP-dsRNA 14dpi-B | 1 | 0.016980232 | 0.178006274 | 0.159320078 | 0.071297732 | 0.010452559 | 0.8946 |
| Ovary-GFP-dsRNA 14dpi-C | 1 | 0.01167851 | 0.204475515 | 0.846745312 | 0.024860515 | 0.046391362 | 1.5151 |
| Intestine-*AjSRα*-dsRNA 7dpi-A | 1 | 0.027969533 | 0.346277367 | 0.747424624 | 0.01674646 | 0.018971795 | 1.0639 |
| Intestine-*AjSRα*-dsRNA 7dpi-B | 1 | 0.014377932 | 0.244855074 | 0.476318999 | 0.014377932 | 0.014377932 | 0.8537 |
| Intestine-*AjSRα*-dsRNA 7dpi-C | 1 | 0.013602353 | 0.320856474 | 0.697371833 | 0.010896435 | 0.010821168 | 0.8161 |
| Intestine-GFP-dsRNA 7dpi-A | 1 | 0.023195681 | 0.233258248 | 0.456915725 | 0.016980232 | 0.020333466 | 0.9605 |
| Intestine-GFP-dsRNA 7dpi-B | 1 | 0.024688791 | 0.423372656 | 0.438302861 | 0.027776334 | 0.027584469 | 1.1602 |
| Intestine-GFP-dsRNA 7dpi-C | 1 | 0.041521432 | 0.742261785 | 0.547146851 | 0.05041511 | 0.041521432 | 1.5767 |
| Ovary-*AjSRα*-dsRNA 7dpi-A | 1 | 0.159320078 | 0.083620472 | 0.888842681 | 0.090873282 | 0.10153155 | 2.5781 |
| Ovary-*AjSRα*-dsRNA 7dpi-B | 1 | 0.092141826 | 0.032352029 | 0.650670928 | 0.042393885 | 0.057511728 | 1.7104 |
| Ovary-*AjSRα*-dsRNA 7dpi-C | 1 | 0.158219574 | 0.032803646 | 0.641712949 | 0.055168937 | 0.054787858 | 1.7986 |
| Ovary-GFP-dsRNA 7dpi-A | 1 | 0.029977004 | 0.047366143 | 0.510506063 | 0.046070913 | 0.047366143 | 1.5658 |
| Ovary-GFP-dsRNA 7dpi-B | 1 | 0.049036506 | 0.024518253 | 0.327598351 | 0.036651092 | 0.036906021 | 1.2435 |
| Ovary-GFP-dsRNA 7dpi-C | 1 | 0.051118879 | 0.0625 | 0.702222438 | 0.077481731 | 0.075362989 | 2.1688 |
| Intestine-*AjSRα*-dsRNA 3dpi-A | 1 | 0.084202099 | 0.083042863 | 0.213158723 | 0.017217267 | 0.114228931 | 1.2264 |
| Intestine-*AjSRα*-dsRNA 3dpi-B | 1 | 0.115823508 | 0.025382887 | 0.146604369 | 0.010821168 | 0.016862941 | 0.6164 |
| Intestine-*AjSRα*-dsRNA 3dpi-C | 1 | 0.097395572 | 0.008974206 | 0.037681495 | 0.002668047 | 0.008668512 | 0.2619 |
| Intestine-GFP-dsRNA 3dpi-A | 1 | 0.088388348 | 0.007921558 | 0.112656308 | 0.007442484 | 0.012516717 | 0.4878 |
| Intestine-GFP-dsRNA 3dpi-B | 1 | 0.127626516 | 0.007391075 | 0.170755032 | 0.003217152 | 0.007704943 | 0.3887 |
| Intestine-GFP-dsRNA 3dpi-C | 1 | 0.120742041 | 0.00786684 | 0.120742041 | 0.005154328 | 0.012430258 | 0.452 |
| Ovary-*AjSRα*-dsRNA 3dpi-A | 1 | 0.081899588 | 0.027016788 | 0.085971364 | 0.006258358 | 0.008789519 | 0.3997 |
| Ovary-*AjSRα*-dsRNA 3dpi-B | 1 | 0.103664943 | 0.074842419 | 0.637280314 | 0.03103414 | 0.031906629 | 1.3584 |
| Ovary-*AjSRα*-dsRNA 3dpi-C | 1 | 0.092141826 | 0.024518253 | 0.118257206 | 0.007651721 | 0.059128603 | 0.733 |
| Ovary-GFP-dsRNA 3dpi-A | 1 | 0.420448208 | 0.061213769 | 0.069830446 | 0.008257953 | 0.012430258 | 0.4435 |
| Ovary-GFP-dsRNA 3dpi-B | 1 | 0.040666933 | 0.052192995 | 0.360982299 | 0.007340021 | 0.015409886 | 0.6851 |
| Ovary-GFP-dsRNA 3dpi-C | 1 | 0.041234622 | 0.081899588 | 0.376311687 | 0.00537321 | 0.006992383 | 0.5225 |
|  |  |  |  |  |  |  |  |
| M<1.5 | **1.407** | 1.988 | 1.885 | **1.42** | **1.485** | **1.431** |  |
